# Etoposide-resistance in a neuroblastoma model cell line is associated with 13q14.3 mono-allelic deletion and miRNA-15a/16-1 down-regulation

**DOI:** 10.1038/s41598-018-32195-7

**Published:** 2018-09-13

**Authors:** Barbara Marengo, Paola Monti, Mariangela Miele, Paola Menichini, Laura Ottaggio, Giorgia Foggetti, Alessandra Pulliero, Alberto Izzotti, Andrea Speciale, Ombretta Garbarino, Nicola Traverso, Gilberto Fronza, Cinzia Domenicotti

**Affiliations:** 1 Department of Experimental Medicine, General Pathology Section, University of Genova, Genova, Italy; 2 UOC Mutagenesis and Oncologic Prevention, IRCCS Ospedale Policlinico San Martino, Genova, Italy; 3 Yale Cancer Center, Yale University School of Medicine, New Haven, Connecticut USA; 4 Department of Health Sciences, University of Genova, Genova, Italy

## Abstract

Drug resistance is the major obstacle in successfully treating high-risk neuroblastoma. The aim of this study was to investigate the basis of etoposide-resistance in neuroblastoma. To this end, a MYCN-amplified neuroblastoma cell line (HTLA-230) was treated with increasing etoposide concentrations and an etoposide-resistant cell line (HTLA-ER) was obtained. HTLA-ER cells, following etoposide exposure, evaded apoptosis by altering Bax/Bcl2 ratio. While both cell populations shared a homozygous *TP53* mutation encoding a partially-functioning protein, a mono-allelic deletion of 13q14.3 locus, where the P53 inducible miRNAs 15a/16-1 are located, and the consequent miRNA down-regulation were detected only in HTLA-ER cells. This event correlated with BMI-1 oncoprotein up-regulation which caused a decrease in p16 tumor suppressor content and a metabolic adaptation of HTLA-ER cells. These results, taken collectively, highlight the role of miRNAs 15a/16-1 as markers of chemoresistance.

## Introduction

Neuroblastoma (NB) is one of the most common extra-cranial solid tumors in childhood and it is characterized by high clinical and biological heterogeneity^1,2^. Among the genetic changes most frequently associated with the aggressive cancer phenotype, the amplification of the MYCN proto-oncogene is an important predictor of high-risk NB^3^. Although most high-risk NB patients initially respond to therapy, a majority of these patients will relapse with treatment-resistant disease. It has been found that approximately 50% of relapsed NBs are associated with the inactivation of the *TP53* tumor-suppressor gene pathways^4^.

The loss of function of the P53 protein may derive either from the mutations of the *TP53* gene^5^, the interaction of P53 with its endogenous inhibitor MDM2^6^, or from the transcriptional and/or post-transcriptional regulation of P53 and P53-dependent genes^7^.

In NB, *TP53* mutations are rare at diagnosis^8^ but P53 inactivation occurs relatively often (~50%) following therapeutic treatment^9^. However, the molecular mechanisms leading to P53 impairment in treatment-resistant diseases have not yet been elucidated. In this context, we have recently demonstrated that HTLA-230, a MYCN-amplified human NB cell line chronically treated with the clinically-used drug etoposide^10^, developed etoposide-resistance and also acquired a multi-drug resistance (MDR) phenotype, thus becoming able to efficiently repair DNA damage and evade apoptosis^11^. Since apoptotic failure, a critical hallmark of cancer^12^, is often determined by the loss of the tumor suppressor activity of P53, herein we initiated the investigation of the role of the P53 pathway in the acquisition of the MDR phenotype.

In recent years, a key role in the acquisition of chemoresistance has been attributed specifically to micro-RNAs (miRNAs^13,14^), which are a family of small non-coding RNAs that have been demonstrated to regulate multiple mechanisms such as drug efflux, drug metabolism, DNA methylation and repair and apoptosis^15^.

In NB, miRNAs have been identified to be down- or up-regulated and associated with MYCN amplification and chemoresistance^13,16^. Interestingly, several miRNAs are able to modulate P53 expression and P53 itself is able to regulate the expression of several miRNAs^17^. Therefore, in the present study, our attention was extended to the involvement of the P53-miRNA network in the observed chemoresistance.

## Results

### Acute etoposide treatment does not modify the mitotic index or the Bax/Bcl2 ratio of HTLA-ER cells

**Figure 1:**
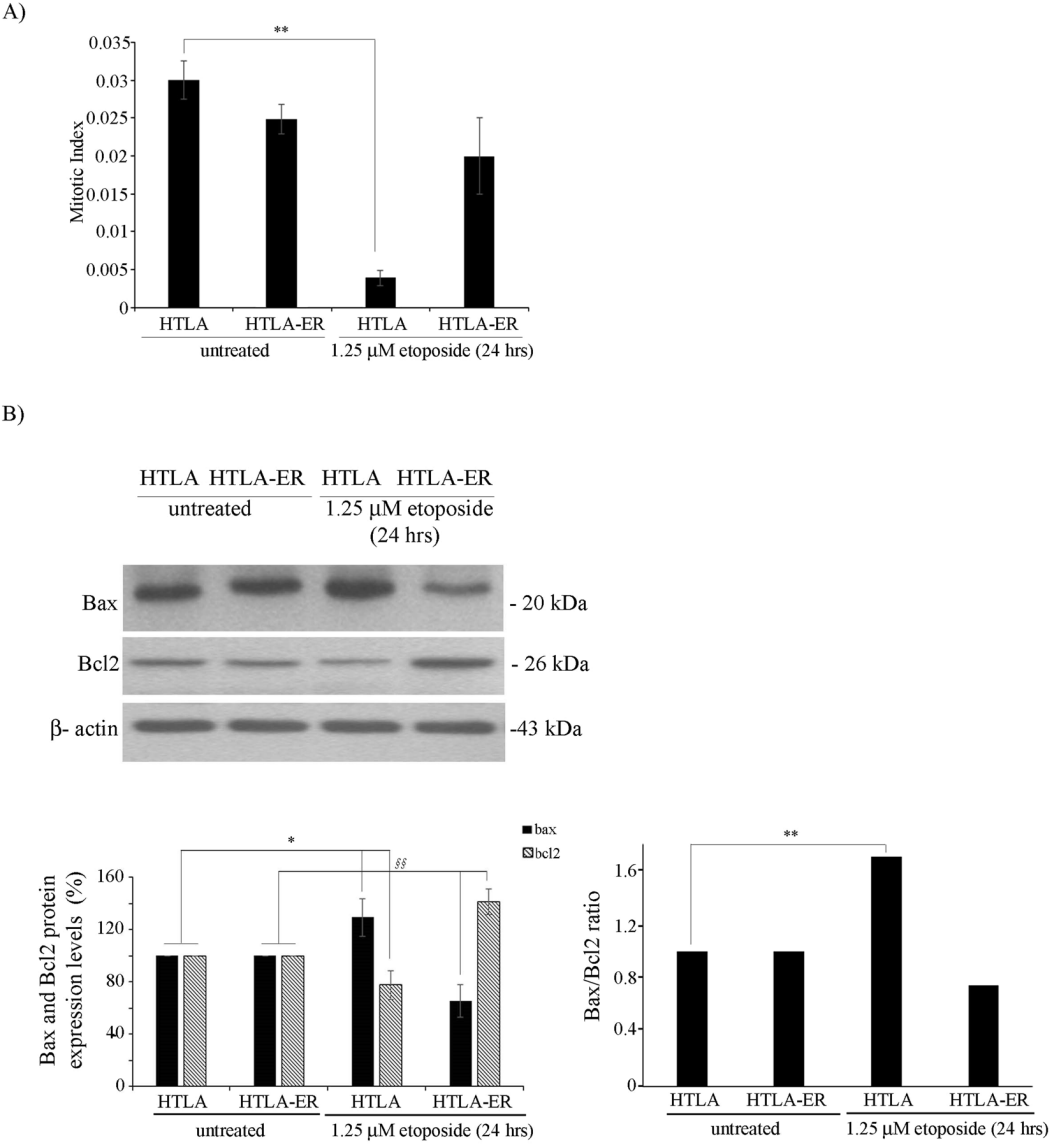
The mitotic index of HTLA-ER cells and their Bax/Bcl2 ratio were not modified by acute etoposide exposure. (**A**) Mitotic index of HTLA-230 and HTLA-ER cells untreated or treated for 24 hrs with 1.25 μM etoposide. Histograms summarize quantitative data of means ± S.D. of four independent experiments per experimental condition (at least 4 × 10^3^ cells per experimental condition were counted) **p < *0.01* vs. untreated HTLA-230 cells. (**B**) Protein levels of Bax and Bcl2 in HTLA-230 and HTLA-ER cells untreated or treated for 24 hrs with 1.25 μM etoposide. Immunoblots are representative of three independent experiments with essentially similar results. β-Actin is the internal loading control. The histograms on the left summarize quantitative data of protein level means, normalized to β-actin expression ± S.E.M of three independent experiments. The histograms on the right summarize quantitative data of Bax/Bcl2 ratio means ± S.E.M of three independent experiments. *p < *0.05* vs. untreated HTLA-230 cells; **p < *0.01* vs. untreated HTLA-230 cells; ^§§^*p* < *0.01* vs. untreated HTLA-ER cells.

We have recently demonstrated that acute etoposide exposure induced DNA damage, apoptosis and a decrease in the proliferation rate in HTLA-230 cells but not in the etoposide-resistant ones^11^. The decrease in the proliferation rate of HTLA-230 cells after acute etoposide treatment was confirmed by mitotic index analysis. As shown in Fig. 1A, etoposide reduced the mitotic index of HTLA parental cells by 87% while the same treatment did not significantly affect the replicative ability of etoposide-resistant cells (HTLA-ER).

Considering the different effects induced by etoposide on the two cell populations, we hypothesized that the acquisition of resistance could be due to changes in the expression of pro- and anti-apoptotic proteins. Immunoblot analysis showed that, following etoposide exposure, Bax levels were increased by 25% in HTLA parental cells and decreased by 35% in HTLA-ER in comparison with the untreated cells (Fig. 1B, upper and left lower panel and Fig. 1 supplementary).

**Figure 2:**
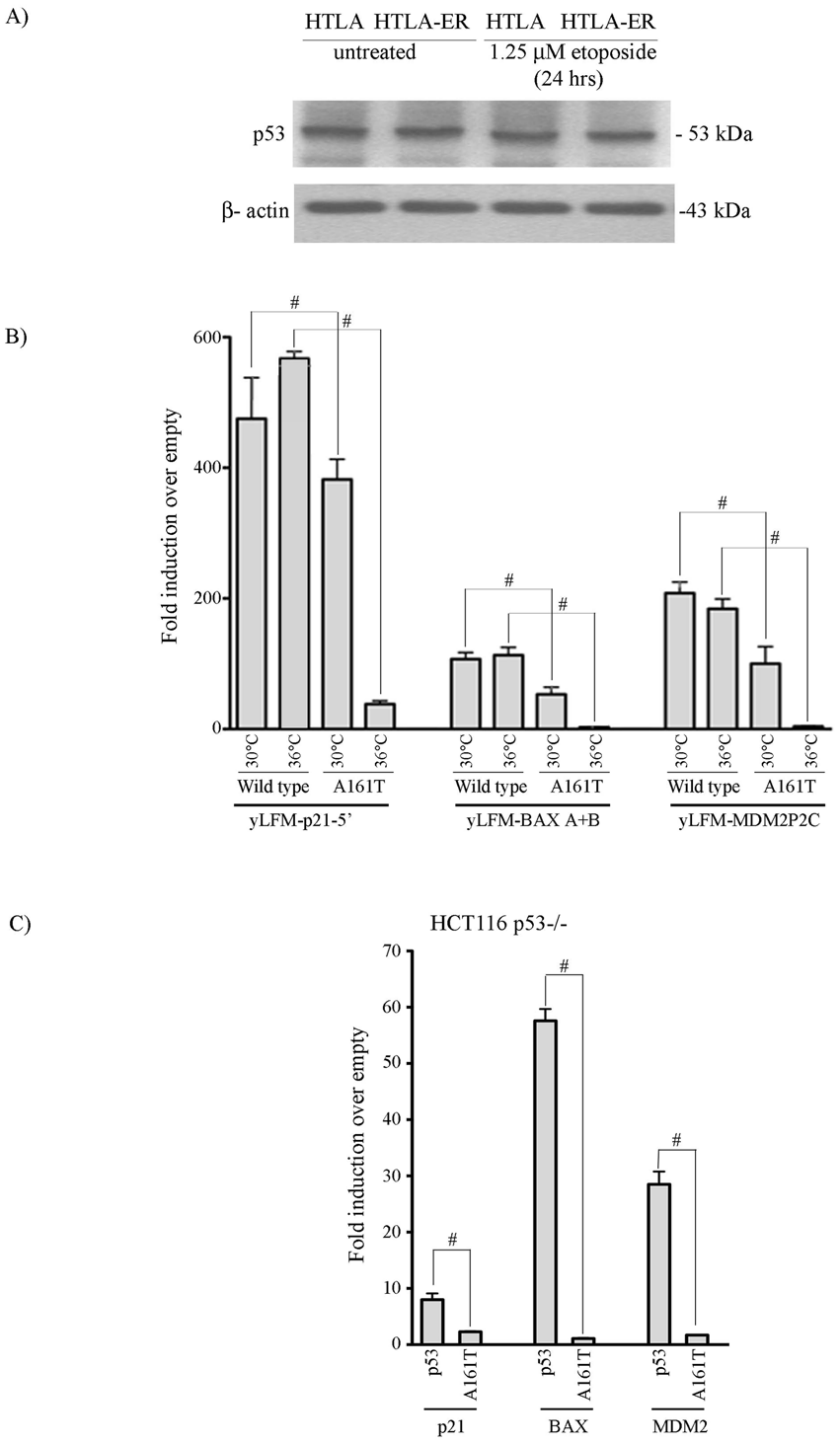
Parental and HTLA-ER cells express a non-inducible P53 protein carrying the homozygous *TP53* missense mutation A161T. (**A**) Protein levels of P53 in HTLA-230 and HTLA-ER cells untreated or treated for 24 hrs with 1.25 μM etoposide. Immunoblots are representative of three independent experiments with essentially similar results. β-Actin is the internal loading control. (**B**) Transactivation ability of wild-type and mutant (A161T) P53 proteins in yLFM-P21-5′, yLFM-BAX A + B and yLFM-MDM2P2C yeast strains. The transactivation ability was determined at two different temperatures (30 °C and 36 °C) using a constitutive expression of P53 proteins (ADH1 promoter). Presented data are the fold of induction over empty vector (pRS315) and standard deviation of four biological replicates. ^#^*p* < *0.001* vs. wild-type P53. (**C**) Transactivation ability of wild-type and mutant (A161T) P53 proteins on reporter constructs (P21, BAX and MDM2) in HCT116 *TP53*^−/−^ human cells. Renilla luciferase was used to normalize transfection efficiencies. Data are expressed as fold of induction relative to the results obtained with an empty vector (pCIneo). Presented data are the average and standard deviations of three biological replicates. ^#^*p* < *0.001* vs. wild-type P53.

In addition, a significant reduction in the Bcl2 level was observed in etoposide-treated HTLA parental cells in respect to HTLA-ER cells whose Bcl2 level was in fact enhanced after etoposide exposure (Fig. 1B, upper and left lower panel and Fig. 1 supplementary). The expression levels of the pro-apoptotic PUMA protein (α and β isoforms), known to interact with Bcl2-family members, were also analyzed and were found unchanged under any of the treatment conditions (Fig. 2 supplementary). In conclusion, the marked increase of Bax/Bcl2 ratio in etoposide-treated parental cells (Fig. 1B, right panel) could explain their propensity to undergo apoptosis in contrast with etoposide-treated HTLA-ER cells.

### HTLA-ER and parental cells have a common homozygous *TP53* missense mutation, not responsible *per se* for the acquisition of chemoresistance

**Figure 3:**
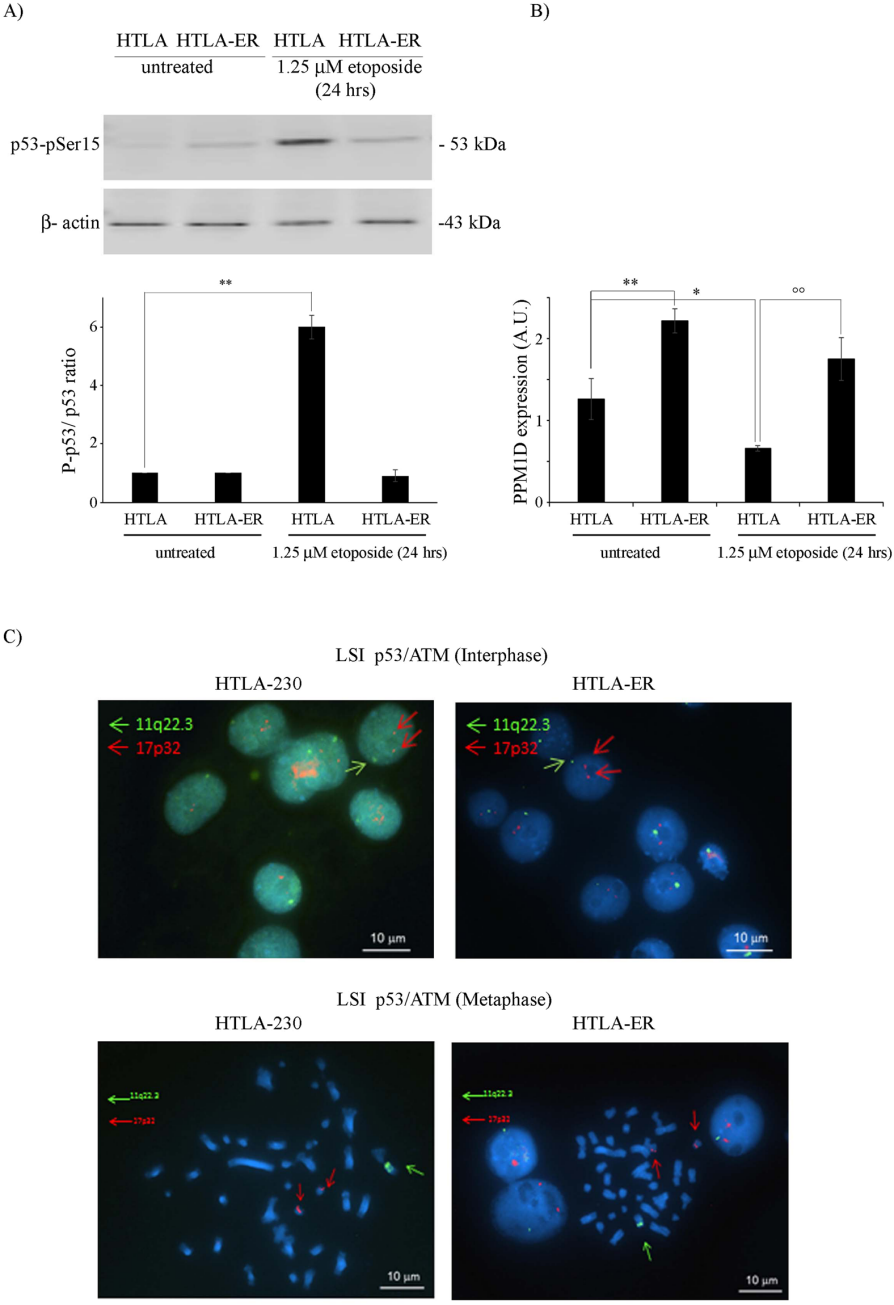
P53 Ser15 phosphorylation is detected only in etoposide-treated HTLA parental cells and is associated with PPM1D up-regulation. (**A**) Protein levels of phospho-(Ser15)-P53 in HTLA-230 and HTLA-ER cells untreated or treated for 24 hrs with 1.25 μM etoposide. Immunoblots are representative of three independent experiments with essentially similar results. β-Actin is the internal loading control. Histograms summarize quantitative data of phospho-P53/P53 ratio means ± S.E.M of three independent experiments. **p < *0.01* vs. untreated HTLA cells. (**B**) RT-PCR analysis of PPM1D/Wip1 in HTLA-230 and HTLA-ER cells untreated or treated for 24 hrs with 1.25 μM etoposide. Histograms summarize quantitative data of PPM1D normalized with means ± S.E.M of three independent experiments. *p < *0.05* vs. untreated HTLA cells; **p < *0.01* vs. untreated HTLA cells; °°*p* < *0.01* vs. 1.25 µM etoposide-treated HTLA cells. (**C**) FISH analysis of HTLA-230 and HTLA-ER cells: upper panels) nuclei of HTLA-230 and HTLA-ER cells with two TP53 and one ATM signals; lower panels) metaphase FISH analysis of HTLA-230 and HTLA-ER cells with two chromosomes 17 displaying one *TP53* signal each and one chromosome 11 displaying ATM signal.

**Figure 4:**
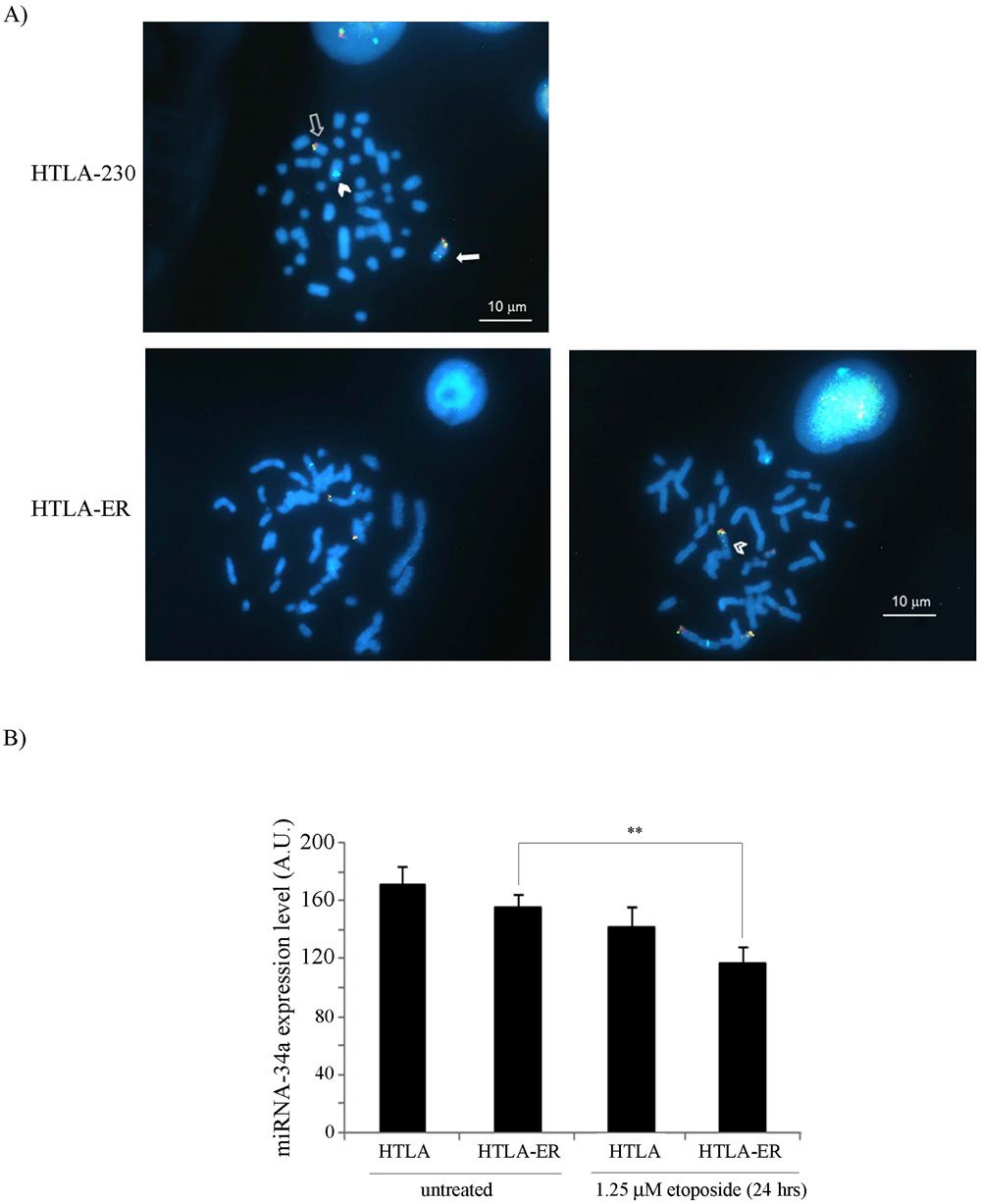
1p36 allelic loss is not observed in parental and HTLA-ER cells and miRNA-34a levels are reduced in both cell populations following etoposide treatment. (**A**) FISH analysis of HTLA-230 cells and HTLA-ER cells. Upper and lower left panels: metaphase of both HTLA-230 and HTLA-ER cells displays one normal chromosome 1 (close arrow) one 1 p arm derivative (close arrowhead) and 1 q arm derivative (open arrow); lower right panel: metaphase of HTLA-ER cells displaying one additional 1 p arm derivative (open arrowhead). (**B**) Expression levels of miRNA-34a in HTLA-230 and HTLA-ER cells untreated or treated for 24 hrs with 1.25 μM etoposide. Histograms summarize quantitative data of means ± S.E.M of three independent experiments. **p < *0.01* vs. untreated HTLA-ER cells.

Since P53 protein plays a key role in the cellular response to genotoxic stress, as well as in the induction and repression of Bax and Bcl-2 respectively^4,18^, its modulation, following etoposide treatment, was investigated in both cell lines. Firstly, gene array analyses revealed that the *TP53* mRNA was overexpressed 7.5 fold in HTLA-ER cells in respect to parental ones (Fig. 3 supplementary). However, immunoblot analysis showed that the two cell populations had a similar high amount of P53 protein which was not inducible by etoposide treatment (Fig. 2A and Fig. 1 supplementary). Moreover, also the level of MDM2 protein, the endogenous inhibitor of P53, was similar and not modified by etoposide treatment in both cell populations (Fig. 4A supplementary), in agreement with the lack of MDM2 amplification revealed by cytogenetic analysis (Fig. 4B supplementary).

The presence of the high amount of P53 protein prompted us to investigate the status of the *TP53* locus. Functional Analysis of Separated Alleles in Yeast (FASAY)^19^ and DNA sequencing (see Materials and Methods) revealed that both cell lines carried a single homozygous *TP53* missense mutation at codon 161 that causes the substitution of Alanine with Threonine (GCC > ACC, A161T).

**Figure 5:**
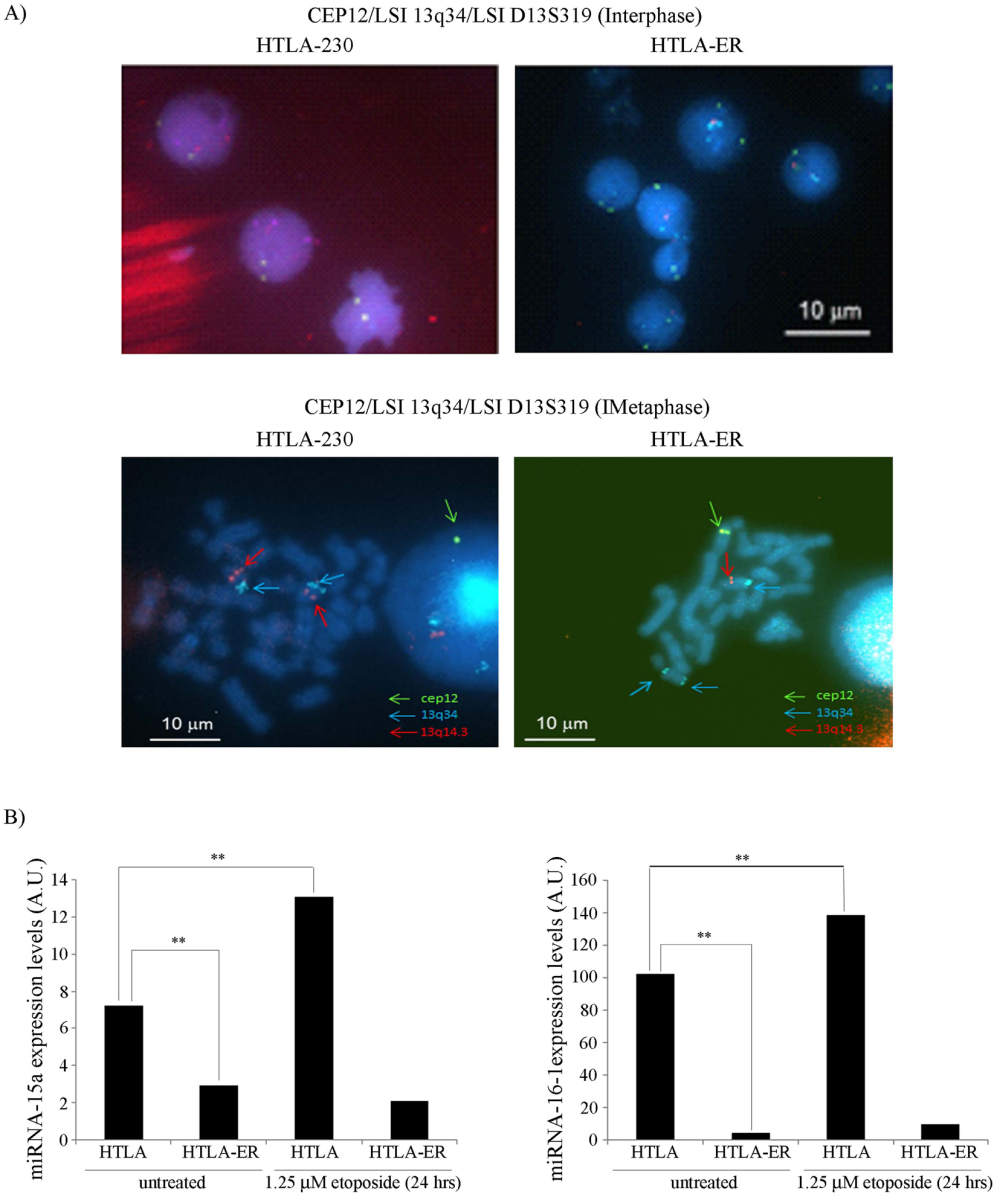
HTLA-ER cells have a deletion at the 13q14.3 locus which is associated with decreased levels of miRNAs 15a/16-1 in respect to parental cells. (**A**) FISH analysis of HTLA-230 and HTLA-ER cells: Upper panels: nuclei of HTLA-230 cells with two CEP12, two 13q34 and two D13S319 signals; nuclei of HTLA-ER cells with two CEP12, three 13q34 and one D13S319 signals. Lower panels: metaphase of HTLA-230 cells with two chromosomes 13 displaying 13q34 and D13S319 signals; metaphase of HTLA-ER cells with one chromosome 13 displaying 13q34 and D13S319 signals, one rearranged chromosome 13 displaying two 13q34, and one chromosome 12 displaying cep 12signal. (**B**) Expression levels of miRNA-15a (left panel) and miRNA-16 (right panel) in HTLA-230 and HTLA-ER cells untreated or treated for 24 hrs with 1.25 μM etoposide. Histograms summarize quantitative data of means ± S.E.M of three independent experiments. **p < *0.01* vs. untreated HTLA-230 cells.

**Figure 6:**
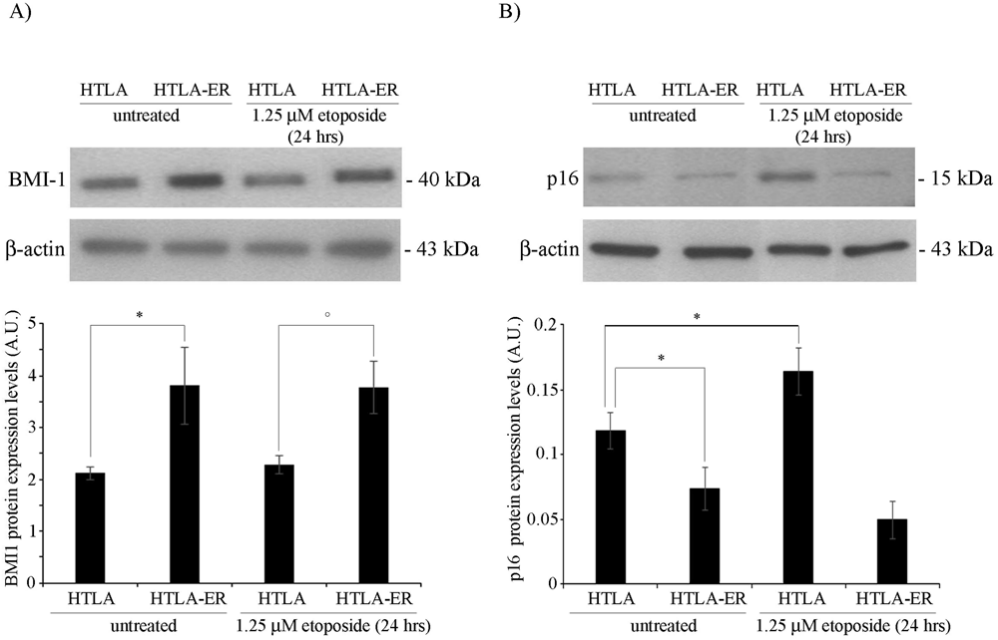
BMI-1 overexpression with consequent p16 down-regulation is found in HTLA-ER cells in respect to parental cells. (**A**) Protein levels of BMI-1 in HTLA-230 and HTLA-ER cells untreated or treated for 24 hrs with 1.25 μM etoposide. Immunoblots are representative of three independent experiments with essentially similar results. β-Actin is the internal loading control. Histograms summarize quantitative data of protein expression levels means, normalized to β-actin expression ± S.E.M of three independent experiments. *p < *0.05* vs. untreated HTLA-230 cells; °*p* < *0.05* vs. 1.25 µM etoposide-treated HTLA-230 cells. (**B**) Protein levels of p16 in HTLA-230 and HTLA-ER cells untreated or treated for 24 hrs with 1.25 μM etoposide. Immunoblots are representative of three independent experiments with essentially similar results. β-Actin is the internal loading control. Histograms summarize quantitative data of protein expression levels means, normalized to β-actin expression ± S.E.M of three independent experiments. *p < *0.05* vs. untreated HTLA-230 cells.

The functional characterization of the identified *TP53* mutation showed that the P53 A161T is temperature-sensitive since, when analyzed with a quantitative yeast functional assay^20–22^, it retained a high transactivation ability at 30 °C but not at 36 °C (Fig. 2B). Such heterogeneity in transactivation was not due to temperature-dependent differences in protein steady-state levels (Fig. 5A supplementary and Fig. 6A supplementary). When expressed in HCT116 (*TP53*^−/−^) mammalian cells, the P53 A161T mutant confirmed the yeast-based data (Fig. 2C, Fig. 5B supplementary and Fig. 6B supplementary).

Therefore, since the same mutation was found in both cell populations, the acquisition of chemoresistance of HTLA-ER is not due to the appearance of a *TP53* mutation in these cells.

### Acute etoposide treatment induced P53 Ser15 phosphorylation in HTLA-230 cells but not in HTLA-ER cells

Considering that P53 is phosphorylated on Ser15 following DNA damage^23^, the phosphorylation status of this site was investigated. As shown in Fig. 3A and Fig. 1 supplementary, etoposide treatment markedly stimulated P53 Ser15 phosphorylation only in the parental cells. Given that the status of Ser15 phosphorylation is regulated by several phosphatases and kinases, their role was taken into consideration. In this context, a two-fold increase in the expression of PPM1D/Wip1, the major phosphatase involved in this process^24^, was found in both untreated and etoposide-treated HTLA-ER cells in respect to the parental cells (Fig. 3B). Moreover, etoposide treatment reduced the expression of PPM1D in HTLA-230 cells by 50% while no significant changes were observed in ER cells (Fig. 3B).

The potential contribution of kinases involved in the P53 pathway, namely ATM, ATR and CHK1^25^, was firstly investigated by microarray analysis that showed a similar expression of the three kinases in parental and in HTLA-ER cell lines (Fig. 3 supplementary). These results are in agreement with the cytogenetic analysis revealing that both cell lines showed nuclei with a single signal at 11q22.3 ATM locus (Fig. 3C). As CHK1 locus maps onto 11q24.2, we can suppose that also this locus was present in a single copy. Moreover, both cell lines showed two chromosomes 17 displaying 17p13 *TP53* locus indicating also that no *TP53* deletion was present (Fig. 3C).

Therefore, we can hypothesize that the different status of P53 Ser15 phosphorylation in parental and resistant cells is due to the differences in the PPM1D phosphatase expression and not to an altered expression of the three main kinases involved in this regulation.

### HTLA-ER cells have a monoallelic deletion at 13q14.3 locus which is accompanied by reduced levels of miRNAs 15a/16-1

It has been reported that, in response to stress conditions, P53 induces the expression of several miRNAs including miRNA-34a^26^ and the miRNA-15a/miRNA-16-1 cluster, the latter being able to reduce the level of Bcl2 and trigger cell death^27^.

To better investigate the effect of the difference in P53 Ser15 phoshorylation found in HTLA-230 and HTLA-ER cells on P53-miRNA network, the involvement of miRNA-34a and miRNA-15a/miRNA-16-1 was analyzed.

MiRNA-34a was reduced in both cell populations after etoposide exposure in comparison with untreated ones (Fig. 4B). Considering that a balanced translocation t(1;17)(p36;q21) involving the 1p36 microdeletion region where miRNA-34a maps has already been demonstrated in HTLA-230 parental cells^28^, the integrity of 1p36 chromosomal region was investigated also in HTLA-ER cells. Our FISH analysis with a probe for 1p36 region revealed the presence of the same chromosome 1 rearrangement in both cell populations (Fig. 4A) confirming that the described t(1;17)(p36;q21) translocation, present in both cell populations, was indeed not associated with a loss of the 1p36 region. Moreover, at least 43% of HTLA-ER cells also showed a duplication of the 1p derivative bearing the 1p36 region (Fig. 4A right panel), indicating the presence of two different subclones.

Similarly, the involvement of miRNAs 15a/16-1 was analyzed by checking the integrity of the chromosomal region 13q14.3 where they are located. The deletion of this region is reported in several malignancies and it has been associated with a loss of tumor suppressor function^29^. As reported in Fig. 5A, interphase FISH analysis showed the presence of two 13q14.3 and 13q34 (used as control marker) loci in the nuclei of parental cells, while it revealed only one 13q14.3 locus and three 13q34 loci in HTLA-ER cells. Moreover, metaphase FISH analysis showed two normal chromosomes 13, each containing 13q14.3 and 13q34 loci, in HTLA-230 cells and one normal and one rearranged chromosome 13, carrying a del(13q14.3) and a dup(13q34), in HTLA-ER cells (Fig. 5A).

In agreement with the observed deletion at the 13q14.3 locus, untreated HTLA-ER cells were characterized by a 70% (Fig. 5B, left panel) and 100% (Fig. 5B, right panel) reduction of miRNA-15a and of miRNA-16-1 expression, respectively, in comparison to untreated parental cells. Moreover, while in parental cells the expression of miRNA-15a and miRNA-16-1 were increased following etoposide treatment by 100% and 44%, respectively, in HTLA-ER cells, the expression level of miRNA-15a and miRNA-16-1 remained unchanged (Fig. 5B). Since both cell lines contained a partially active P53 mutant, the regulation of miRNAs in HTLA-230 cells can be considered P53 independent. In conclusion, these results clearly indicate that HTLA-ER cells have a monoallelic deletion at the 13q14.3 locus, which is accompanied by reduced levels of miRNAs 15a/16-1 that remained unchanged after etoposide treatment.

### Reduced levels of miRNA-15a and miRNA-16-1 in HTLA-ER cells correlate with the increased expression of BMI-1

The reduced expression of miRNA-15a and miRNA-16-1, as well as the lack of their induction following etoposide treatment, can only be partially responsible for the increase in Bcl2 levels found in HTLA-ER cells. In fact, both untreated HTLA-230 and HTLA-ER cells have comparable Bcl2 levels (Fig. 1B) while the level of miRNAs 15a/16-1 was significantly lower in HTLA-ER than in HTLA-230 cells, regardless of etoposide treatment (Fig. 5B).

BMI-1 protein (B lymphoma Mo-MLV insertion region 1), which is a direct target of miRNA-15a and miRNA-16-1^30^, has been reported to be essential for the tumorigenicity of neuroblastoma cells^31^ and to mediate the up-regulation of Bcl2^32^. In accordance, microarray analysis revealed that the expression of BMI-1 increased 5-fold in HTLA-ER in respect to parental cells (Fig. 3 supplementary). Moreover, the BMI-1 overexpression was directly related to the higher levels (+80%) of BMI-1 protein in both untreated and etoposide-treated HTLA-ER in respect to parental cells (Fig. 6A and Fig. 1 supplementary). However, no changes in BMI-1 following etoposide treatment were observed in both cell lines, indicating a Bcl2 modulation not strictly dependent on BMI-1.

Lastly, as BMI-1 is able to regulate cell proliferation^33^ also by down regulating p16^34^, the levels of this tumor suppressor was analyzed. As reported in Fig. 6B and Fig. 1 supplementary, p16 levels were reduced by 35% in HTLA-ER in respect to parental cells. In addition, p16 content was increased by 45% in parental cells following etoposide treatment while it remained unchanged in HTLA-ER cells (Fig. 6B and Fig. 1 supplementary). Altogether, the data pointed out the down-regulation of miRNA-15a and miRNA-16-1 as the major trigger of HTLA-ER chemoresistance by enhancing the basal level of BMI-1 expression, with important consequences on downstream targets (e.g. p16).

## Discussion

Chemotherapy plays a crucial role in the treatment of cancer but its clinical success is often limited by the acquisition of a therapy-induced resistance^35^. In this regard, we have recently demonstrated that long-term exposure to etoposide, a common clinically-used chemotherapeutic drug, is able to select a population of drug-resistant NB cells (HTLA-ER cells) since they are unable to undergo apoptosis^11^.

In this article, we show that drug-sensitive HTLA-230 cells respond to etoposide treatment by decreasing their replicative activity while HTLA-ER cells do not significantly change the mitotic index of untreated cells (Fig. 1). Taking these different cellular responses into account, we have hypothesized that the resistant phenotype of HTLA-ER cells might be the consequence of a different balance between the expression of pro- and anti-apoptotic proteins.

**Figure 7:**
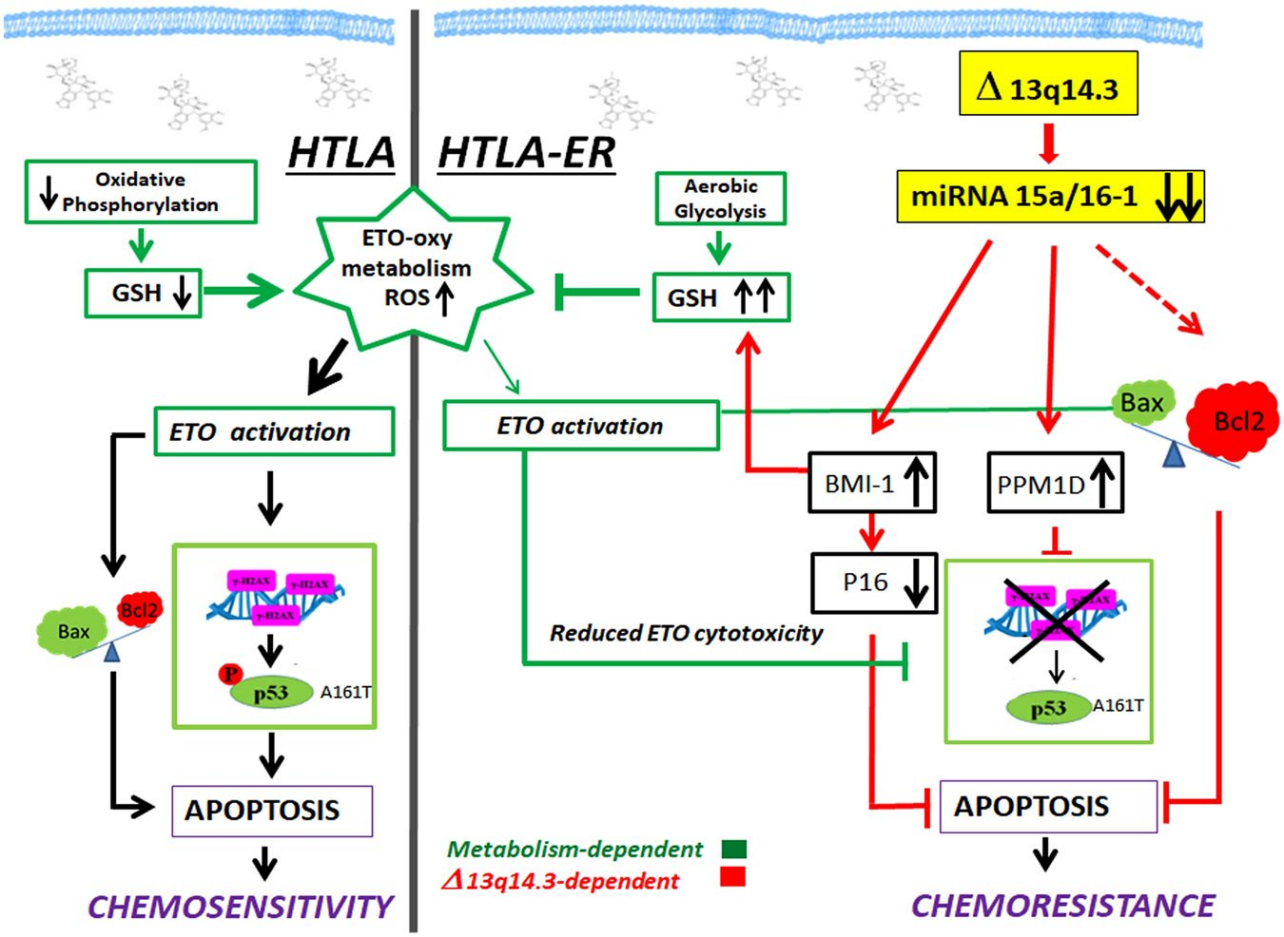
Molecular mechanisms underlying the chemoresistance of HTLA-ER cells. This figure illustrates the observed molecular mechanisms underlying chemoresistance of HTLA-ER cells and the events leading to apoptosis in etoposide sensitive HTLA parental cells. Left panel: Short-term treatment with etoposide of HTLA-230 cells reduces oxidative phosphorylation and decreases glutathione (GSH) levels inducing reactive oxygen species (ROS) overproduction, thus leading to DNA damage (H2AX). Consequently, etoposide-induced genotoxic stress increases pro-apoptotic Bax, reduces anti-apoptotic Bcl2 and stimulates P53-Ser15 phosphorylation, two events leading to apoptosis and chemosensitivity. Right panel: HTLA-ER cells are able to efficiently counteract etoposide-induced ROS production by maintaining an efficient aerobic metabolism and increasing GSH levels. Long-term treatment with etoposide causes a deletion of the 13q14.3 locus and the consequent downregulation of miRNAs 15a/16-1, stimulating several pro-survival signals which contribute to inducing chemoresistance.

In agreement with literature, demonstrating that an imbalance of the Bax/Bcl2 ratio in favor of Bcl2 contributes to rendering cancer cells more resistant to apoptosis^36^, HTLA-ER cells, after short-term etoposide exposure, show an increased amount of Bcl2 that efficiently counteracts the pro-apoptotic function of BAX and/or PUMA (Figs 1 and 2 supplementary)^37^ becoming unable to undergo apoptosis (Fig. 7).

Focusing our attention on P53, a critical modulator of Bcl2 family members^4,18^, we have found that: (i) *TP53* mRNA overexpression in HTLA-ER cells is not accompanied by an increase in the protein level (Figs 2 and 3 supplementary); (ii) the amount of P53 protein is high and not inducible in either cell line (Fig. 2); and (iii) MDM2, the primary negative regulator of P53 (Fig. 4 supplementary)^38^, is not amplified in either cell population. Moreover, our results show that HTLA-230 and HTLA-ER cells have a homozygous *TP53* mutation (A161T) which encodes for a P53 protein with partial transactivation activity (Figs 2 and 5 supplementary).

Since the acquisition of chemoresistance in HTLA-ER cells is not due to the alterations of the *TP53* gene, we hypothesized that post-translational modifications, important for P53 activation, might play a crucial role. In this regard, we have found that P53 phosphorylation on Ser15, which is crucial for the activation of P53-dependent responses^39^, is absent in etoposide-treated ER cells (Figs 3 and 7). This event can be correlated to the overexpression of PPM1D/Wip1 (Figs 3 and 7), a key phosphatase able to modulate the P53 phosphorylated status^24^. Interestingly, it has been demonstrated that the high expression of PPM1D is the most likely target of the 17q23gain/amplification in NB and is related to a poor clinical outcome^40^.

The P53-miRNAs network is an important player in regulating chemoresistance^41^ and the miRNA-34a expression is induced by P53^42^. In this context, the 1p36 locus, where miRNA-34a maps, has not been found deleted in either parental or resistant cells in spite of the presence of the balanced t(1;17)(p36;q21) translocation described in HTLA-230 cells^28^. This leads us to exclude the role of miRNA-34a in the acquisition of chemoresistance in HTLA-ER cells. We then focused our attention on miRNA-15a/16-1 which are other important players of chemoresistance and are induced by P53. MiRNAs 15a/16-1 are located on the 13q14.3 locus, and miRNA-16-1 regulates PPM1D expression^43^, leading us to hypothesize a potential role of these miRNAs in the drug-resistance of HTLA-ER cells.

In this direction, we have found that the genetic feature that distinguishes HTLA-ER from parental cells consists of the monoallelic deletion of the 13q14.3 locus in which miRNA-15a and miRNA-16-1 are located (Figs 5 and 7)^29^. It is worth noting that the 13q14.3 locus has frequently been found deleted in tumors^29,44^ but our results demonstrate for the first time that the deletion is present in etoposide-resistant NB cells. In accordance with data from other authors, the down-regulation of miRNA-15a and miRNA-16-1 induces an enhancement of the *TP53* mRNA^27,45^, not associated with a proportional increase of the P53 protein.

It is important to note that in our model the reduced expression of miRNA-15a/16-1 in HTLA-ER cells was not correlated with Bcl2 levels since a Bcl-2 increase was observed in ER cells only after acute etoposide exposure. Therefore, it is possible to hypothesize that other mechanisms are responsible for the up-regulation of Bcl2 following etoposide treatment.

In addition, it has been reported that miRNA-15a and miRNA-16-1 directly modulate the expression of BMI-1 protein which is highly expressed in several kinds of tumors with a poor prognosis^30,31,46^. In this regard, we show that the low level of miRNAs 15a/16-1 in HTLA-ER cells leads to BMI-1 induction which in turn reduces the expression of the tumor-suppressor protein p16 (Figs 6 and 7)^33,47^, thus contributing to HTLA-ER chemoresistance.

In our *in vitro* model of etoposide-resistance, we have previously demonstrated that HTLA-ER cells are characterized by higher levels of glutathione (GSH) and an up-regulation of catalase and superoxide dismutase activity in respect to parental cells: this antioxidant response leads to a lower production of reactive oxygen species (ROS)^11^.

Interestingly, in chemoresistant ovarian cancer cells, it has been demonstrated that BMI-1 expression regulates GSH production^42^. Thus, the high level of BMI-1 in HTLA-ER cells could enhance the GSH content and hamper the pro-oxidant action of the drug, reducing its cytotoxicity (Fig. 7). The intracellular redox state might well contribute to the inhibition of etoposide-induced γ-H2AX expression and P53 Ser15 phosphorylation in HTLA-ER cells (Fig. 7). The increased level of GSH in the resistant NB cells is probably also due to the stimulation of an efficient aerobic metabolism^11^. These findings are consistent with other studies demonstrating that agents depleting GSH, in combination with conventional therapeutics, synergistically improve the efficacy of the treatment of neuroblastoma^11,48^. Lastly, it is also worth noting that, as reported in many drug-resistant cancers^49^, HTLA-ER cells have an increased activity of glutathione S-transferase (GST), which is a phase II detoxification enzyme, catalyzing the conjugation of GSH with electrophilic substrates. In particular, we have previously reported that both GST expression and activity were increased about 2 fold in etoposide treated resistant cells^11^. Here, we found that 43% of HTLA-ER cells showed a duplication of 1p arm (Fig. 4A, right panel), which is known to include the locus of glutatione S-transferase M1, an enzyme belonging to the GST family^50^. Therefore, we can hypothesize that also the duplication of the 1p arm might contribute to the observed chemoresistant phenotype of NB cells.

Summarizing, our results show that chronic treatment with etoposide of a high-risk NB cell line correlated with a monoallelic deletion of the 13q14.3 locus and a marked down-regulation of miRNA-15a/16-1 levels. This leads to the up-regulation of BMI-1 protein with pleiotropic effects such as the activation of GSH-dependent responses which could be involved in the metabolic adaptation of drug resistant NB cells (Fig. 7).

However, further studies are needed to highlight the potential role of these miRNAs as markers of drug resistance and also as therapeutic targets in order to modulate the epigenetic changes supporting NB chemoresistance.

## Materials and Methods

### Cell cultures

#### NB cell culture conditions and treatments

The MYCN-amplified human stage-IV NB cell line, HTLA-230, was obtained from Dr. L. Raffaghello (G. Gaslini Institute, Genoa, Italy). Cytogenetic features of HTLA-230 cell line including 4p MYCN amplification, del(11)t(11;Y), balanced translocation t(1;17)(p36;q21) and dup(11p) have been previously described by Pezzolo *et al*.^28^. The cell line was periodically tested for mycoplasma contamination (Mycoplasma Reagent Set, Aurogene s.p.a, Pavia, Italy). After thawing and eight passages in the culture, cell morphology and proliferation were analyzed. Cells were cultured in RPMI 1640 (Euroclone SpA, Pavia, Italy) supplemented with 10% fetal bovine serum (FBS; Euroclone), 2 mM glutamine (Euroclone), 1% penicillin/streptomycin (Euroclone), 1% sodium pyruvate (Sigma-Aldrich, Sant Louis, Missouri, USA), and 1% of aminoacid solution (Sigma).

The etoposide-resistant cell line (HTLA-ER) was selected by treating HTLA-230 cells for 6 months with increasing concentrations of etoposide^11^.

Parental HTLA-230 and HTLA-ER cells were treated for 24 hrs with 1.25 µM etoposide (Calbiochem, Merck KGaA, Darmstadt, Germany). The stock solutions of etoposide were prepared in DMSO and pilot experiments demonstrated that the final DMSO concentrations did not change any of the cell responses analyzed.

#### Yeast cell culture conditions

*S. Cerevisiae* yeast cells were grown in YPDA medium (1% yeast extract, 2% peptone, 2% dextrose, 200 mg/L adenine) or in a selective medium containing dextrose as a carbon source and adenine (5 mg/L or 200 mg/L) but lacking leucine or tryptophan for the selection of the expression vector (Sigma-Aldrich; BiokarDiagnostics, Allonne, France). All transformations of yeast cells were based on a lithium acetate method^51^.

#### Human colon carcinoma cell culture conditions

HCT116 *TP53*^−/−^ cells (human colon carcinoma) were obtained by Dr. B. Vogelstein (The Johns Hopkins Kimmel Cancer Center, Baltimore, MD). Cells were grown in RPMI containing 10% fetal bovine serum (Euroclone) and maintained at 37 °C in 5% CO_2_ at 100% humidity.

### Mitotic Index

Exponentially-growing HTLA-230 and HTLA-ER cells were seeded 24 hrs before drug treatment on 8-well chamber slides (Thermo Fisher Scientific, Waltham, MA USA) or on 24 × 24 coverslips. After 24 hrs of treatment, cells were fixed in methanol:glacial acetic acid 3:1 for 15 min at 4 °C. After fixation, cells were washed in PBS and counterstained with DAPI for 5 min or with 3% Giemsa Stain for 15 min. Finally, cells were observed using an epifluorescence microscope (Provis AX70, Olympus, Milano, Italy); mitoses and interphases (at least 500 cells per experimental condition) were counted from two independent scores in four independent experiments.

### Immunoblot analysis

#### Immunoblot analysis of NB cell protein extracts

NB cell protein extract was prepared as previously described^52^. Immunoblots were carried out according to standard methods^53^ using rabbit antibodies anti-bax and anti-bcl2 (Abcam, Cambridge, UK), anti-PUMA and anti-BMI1 (Cell Signalling Technology Inc., Danvers, MA, USA), a phospho-p53 Antibody Sampler Kit (Cell Signaling Technology Inc.) and mouse antibodies anti-β-actin (Sigma), anti-MDM2 and anti-p16 (Santa Cruz Biotechnology Inc., Dallas, Texas, USA). Anti-mouse and anti-rabbit secondary antibodies were coupled with horseradish peroxidase (GeHealthcare, Buckinghamshire, UK). Proteins were visualized with an enzyme-linked chemiluminescence detection kit according to the manufacturer’s (GeHealthcare) instructions. Chemiluminescence was monitored by exposure to film and the signals were analyzed under non-saturating conditions with an image densitometer connected to Quantity One software (Bio-Rad Laboratories, Hercules, CA, USA).

#### Immunoblot analysis of yeast and mammalian protein extracts from reporter assays

Yeast extracts were prepared as described^54^. Briefly, yeast cells recovered from plates were resuspended in 100 μl of distilled water and 100 μl of 0.2 M NaOH. After 5 min of incubation at room temperature, the cells were pelleted, resuspended in 50 μl SDS sample buffer (0.06 M TrisHCl, pH 6.8, 5% glycerol, 2% SDS, 4% β-mercaptoethanol, 0.0025% bromophenol blue), boiled for 3 min and pelleted again.

Twenty μl of yeast supernatant and 20 μg of mammalian cell extracts in Passive Lysis Buffer (PLB, Promega, Madison, Wisconsin, U.S.A) from transformation and transfection assays, respectively were loaded on 12% SDS-PAGE using a Biorad MiniProtean apparatus (Bio-Rad) and transferred to a nitrocellulose membrane (GEHealthcare). Detection was carried out with ECL Fast Pico (ECL-1002, Immunological Sciences, Roma, Italy). Chemiluminescence was analyzed by Alliance LD, UVITEC Cambridge (Cambridge, UK).

The following antibodies were employed: anti-p53 (DO-1, sc-126, Santa-Cruz), anti-β-actin (AC-74, Sigma), anti-yeast Phosphoglycerate Kinase 1 (PGK1)(22C5D8, Thermo Fisher Scientific) and a secondary anti-mouse IgG peroxidase conjugate (A9044, Sigma).

### Cytogenetic analysis

#### Interphase nuclei and metaphase preparation

Interphase nuclei were obtained by detaching cells with trypsin EDTA. C-metaphases were obtained by standard procedures, using 0.1 μg/mL colcemid (GIBCO BRL, Thermo Fisher Scientific) for 2 hrs. All samples were then fixed in methanol:glacial acetic acid 3:1, splashed, dried, and aged 2–3 days before hybridization procedures.

#### Fluorescence *In Situ* Hybridization (FISH) analysis

Cytogenetic abnormalities were determined by FISH analysis. Amplification of the MDM2 gene was investigated by using the LSI MDM2/CEP12 FISH probe kit (Abbott-Vysis, Roma, Italy) which included the SpectrumOrange-labeled LSI MDM2 probe that hybridizes the 12q15 locus containing the MDM2 gene and the SpectrumGreen CEP 12 probe that hybridizes the alphoid sequences found within the centromere of chromosome 12 (12p11.1-q11). The following abnormalities including del13q14 (D13S319 probe), del11q22 (ATM probe), del17p13 (TP53 probe) and trisomy 12 (CEP 12 DNA Probe) were tested by using a disease-specific comprehensive probe set (Abbott-Vysis). This probe set included: (i) the SpectrumGreen-labeled LSI ATM probe (732 kb in size) that hybridizes the 11q22.3 locus containing RAB39, CUL5 and EXPH5 genes other than the ATM gene (ii) the SpectrumOrange-labeled LSI *TP53* probe, that hybridizes the 17p13.1 locus containing the complete *TP53* gene (iii) the SpectrumOrange-labeled LSI D13S319 probe which is 135 kb in length and hybridizes the 13q14.3 locus encompassing the DLEU1 locus and (iv) the SpectrumAqua-labeled LSI 13q34 that hybridizes the 13q34 locus used as control. The 1p deletion was investigated using the 1p36 Microdeletion Region Probe (Abbott-Vysis). This probe included (i) LSI p58 (1p36) (SpectrumOrange) (ii) TelVysio 1p (SpectrumGreen) used as control and (iii) LSI 1q25 (SpectrumAqua) used as control. Standard interphase FISH was performed according to the manufacturers’ recommendations. At least 200 nuclei were analyzed for each probe by two independent scores. Metaphases were also analyzed.

### Digital image analysis

Images were acquired using an epifluorescence microscope (as above) equipped with a digital monochrome progressive CCD camera (CV-M4 + CL progressive scan, JAI Corporation, Japan) driven by CytoVision™ system (Applied Imaging, SAN JOSE, CA, USA). DAPI, FITC, TRITC and AQUA images were acquired with selective single-bandpass filters at 100x optical magnification, and merged.

### Gene expression analysis by cDNA microarray

The expression of 18,401 human genes was tested by cDNA microarray. Custom microarrays, made available by the Microarray Department-University of Amsterdam, were used^55,56^. The whole list of spotted genes is available on the website http://www.micro-array.nl/libraries.html. All data is MIAME-compliant as detailed on the MGED Society website (http://www.mged.org/Workgroups/MIAME/miame.html). Microarrays were made available by the Microarray Department of the University of Amsterdam (http://www.micro-array.nl). The gene expression analysis was performed, as previously reported^11^. The call/response rate obtained was >90%, which was used as quality criterion for microarray analysis.

### RNA extraction and reverse transcription

Total RNA was extracted using TRIZOL reagent (LifeTechnologies, Carlsbad, California, USA) according to the manufacturer’s instructions. Total RNA (1 μg) was reverse-transcribed into cDNA by a random hexamer primer and SuperScript™ II Reverse Transcriptase (LifeTechnologies).

### Functional Analysis of Separated Alleles in Yeast (FASAY)

The FASAY assay is used to define the functional status (wild-type or mutant) of a *TP53* allele. This assay exploits the yIG397 *S. Cerevisiae* yeast strain that contains the ADE2 open reading frame under the control of a p53-responsive element (3XRGC); the ADE2 gene is involved in the biosynthetic pathway of adenine. Cells containing wild-type P53 express ADE2 and form white colonies on the plate with a limited amount of adenine (5 mg/L). Conversely, cells containing a mutant P53 (i.e. not able to transactivate the reporter gene) do not express ADE2 and generate small red colonies on the same plates due to an accumulation of a colored intermediate product in the biosynthetic pathway of adenine^19^.

To perform this analysis, cDNA from HTLA-230 and HTLA-ER cells was firstly amplified by using *TP53* specific primers P3 and P4^19^ and Exact Polymerase (5 Prime). The following PCR conditions were used: 1 min of denaturation at 94 °C, 1 min of annealing at 55 °C and 1 min of elongation at 72 °C (30 cycles). An initial heat-activation step at 95 °C for 5 min was required. The yIG397 yeast strain was then co-transformed with unpurified P53 PCR product (10 µl) and pRDI22 HindIII/StuI digested vector (50 ng). In the yeast, the plasmid is re-sealed together with the PCR products by recombination, exploiting the sequence homology at the end of the fragments (Gap Repair Assay)^19^. By analyzing the *TP53* status from a cell line, it is expected that the presence of a non-functional *TP53* allele in heterozygosity generates around 50% of small red colonies. In the case of homozygosity for the presence of a non-functional *TP53* allele, the yeast cells are expected to be all red. The background level of the assay was also evaluated by using PCR amplification (P3-P4) on the pLS76 plasmid template that harbours the wild-type *TP53* cDNA.

The output of the described analysis showed a percentage of red yeast colonies over the total number of transformants (white and red colonies) of 16.9% and 14.2% for parental and HTLA-ER cells, respectively, suggesting the presence of a functional (wild-type) P53 protein (referred at 30 °C).

### Sequencing of *TP53* PCR product

PCR on *TP53* cDNA was performed with primers P1 and P10 (Table 1) that span the entire *TP53* coding sequence. The previously described PCR conditions were used. The purified PCR product (QIAquick PCR purification Kit, Qiagen, Hilden, Germany) was sequenced with primers P5 and P6 (Table 2; BMR Genomics, Padua, Italy). In order to accurately check the sequence, including the 15–20 N-terminal and C-terminal amino acidic residues (i.e. residues that cannot be analyzed for technical reason by the previous analysis), we decided to clone the cDNA of parental HTLA-230 and HTLA-ER cells in a yeast expression vector (pTS-based, TRP1 as selection marker). To this end, the *TP53* cDNA sequence was amplified using a pair of primers that are characterized by a 5′ homology (in bold) with the XhoI/NotI double digested pTS-based vector and by a 3′ homology with *TP53* cDNA (underlined): Nter: 5′- **caagctataccaagcatacaatcaactatctcatatacagttaactcgag**atggaggagccgcagtcagatcctagcgtc-3′; Cter: 5′-**gacataactaattacatgatggtggcggccgctctagaactagtggatcc**tcagtctgagtcaggcccttctgtcttgaa-3′. The pTS vector harboring the cDNA of parental HTLA-230 or HTLA-ER cells was constructed using the previously described Gap Repair Assay (see above). Plasmid DNA was recovered from yeast colonies, expanded in *E. coli*, and checked by digestion. At least two different clones, deriving from PCR amplification of cDNA from parental HTLA-230 or HTLA-ER cells, were verified by DNA sequencing (P5 and P6 primers) (BMR Genomics).

**Table 1:** Sequence of primers used for PCR analyses.

Official Gene Symbol	Primers
*TP 53* (P1)	5′-atggaggagccgcagtcagatcctagcgtc-3′
*TP 53* (P5)	5′-tggccatctacaagcagtca-3′
*TP 53* (P6)	5′-gggcaccaccacactatgtc-3′
*TP 53* (P10)	5′-tcagtctgagtcaggcccttctgtcttgaa-3′
PPM1D	Forward 5′-atttcgctgggcggattttg-3′ Reverse 5′-agtggagcttcgcatagagc-3′

**Table 2:** Sequences of primers and probes used for TaqMan qPCR.

Universal Reverse Primer	Sequence of probes	TaqMan assay I
has-miR-16-1	5′- uagcagcacguaaauauuggcg -3′	4427975AssaYID 000391
has-miR-15a	5′- uagcagcacaucaugguuuaca- 3′	4427975AssayID000390
has-miR-34a	5′-caaucagcaaguauacugcccu-3′	4427975 AssayID002316
RNU38B	5′- TCTCAgTgATgAAAACTTTgTCCAg-3′	4427975ASSAYID 01004

### Construction of mutant *TP53* allele by a two-step PCR mutagenic approach

A pair of complementary 30-mer oligonucleotides (which served as forward and reverse primers) was synthesized with the mutated base adjacent to the central position of the oligonucleotide (A161T forward: 5′-gtccgcgccatgaccatctacaagcagtca-3′; A161T reverse: 5′-tgactgcttgtagatggtcatggcgcggac-3′)^20^. The forward and reverse primers were used in two separate PCR reactions (for conditions see above) and paired with P4 and P3 primers respectively, using pLS76 plasmid as a template. As previously described, homologous recombination *in vivo* in yeast was exploited by using the two PCR products and the pRDI22 HindIII/StuI digested vector. The re-sealed plasmid DNA was recovered from yeast transformants through genomic extraction and expanded in *E. coli*. The presence of the specific *TP53* mutation (GCC > ACC, A161T) was verified at the molecular level by DNA sequencing (BMR Genomics).

### Yeast functional reporter assay

The yLFM-P21-5′, yLFM-BAX A + B and yLFM-MDM2P2C strains were used to quantitatively evaluate the functionality of the P53 mutant (A161T); all strains are isogenic except for the different Response Element (RE) located upstream from the luciferase reporter gene^57^. Briefly, yeast strains were transformed with pLS-based expression vectors (encoding wild-type and mutant P53 from the ADH1 constitutive promoter) along with the empty vector pRS315.

The functional assay was carried out according to the miniaturized protocol previously developed by us^21^. Yeast transformants (selected on 200 mg/L adenine plates) were grown at two different temperatures (30 °C and 37 °C) and then resuspended in 150 μl of water. OD (600 nm) was directly measured in a transparent 96-well plate. The cell suspension (20 μl) was transferred into a white plate and mixed with an equal volume of PLB buffer 2X in order to obtain the lysis of yeast cells. After 15 min of shaking at room temperature, firefly luciferase substrate (20 μl, Bright Glo, Promega) was added. Luciferase activity was measured using a multilabel plate reader (Mithras LB940, Berthold Technologies) and normalized to OD 600 nm. The transactivation ability of the wild-type and mutant P53 proteins was expressed as fold of induction over empty vector (pRS315).

### Mammalian functional reporter assay

Wild-type and mutant (A161T) P53 proteins were expressed by a pCIneo-based vector. The mutant P53 expression vector was first constructed in a yeast pTSG-based plasmid (through SgraI/StuI digestion and subsequent ligation) from the available yeast pLS-based vector. The mammalian pCI-neo plasmid was then obtained from XhoI/NotI double digestion of the pTSG-based vector along with the empty pCI-neo backbone. The presence of the correct sequence was confirmed by sequencing (BMR Genomics).

HCT116 *TP*53^−/−^ cells were seeded in 24-well plates (8 × 10^5^ cells) and transfected using the *Trans*IT-LT1 transfection reagent (Mirus, Milan, Italy) according to the manufacturer’s instructions. The transfection mixture (500 ng/well) contained 250 ng of the different pGL3 promoter-derived p53 reporter plasmids, 200 ng of the expression or empty vector, and 50 ng of the pRL-SV40 control plasmid. Cells were harvested 24 hrs after the transfection, washed with PBS and lysed for 15 min with PLB 1X. Luciferase assays were conducted as previously described^58^.

### Real time PCR analysis

Validation of microarray data was performed by real time-qPCR for PPMD1 gene (Table 1) SYBRGREEN fluorescent tracer was used to identify amplicons whose identity was checked by melting curve analysis. Primer sequences (TIB Molbiol, Italy) were identified according to http://www.ncbi.nlm.nih.gov/tools/primer-blast/database. cDNAs were prepared using Superscript II Reverse Transcription kit (Invitrogen, Milan, Italy). PCR was performed in a Rotor-Gene 3000 Corbett Research, Mortlake, Australia). Each reaction was carried out using 10xPCR buffer, 100 mM dNTPs mix, 50 mM MgCl2, 10 µM primer F, 10 µM primer R, 5 U/μl Platinum® Taq DNA polymerase (Invitrogen), cDNA (diluted 1:10), and SYBRGREEN® (Invitrogen) in a 50-μl reaction volume. The thermal profile consisted of hot-start enzyme activation at 95 °C for 2 min, 45 cycles of PCR at 94 °C for 45 sec (denaturation), at 62 °C for 30 sec (gene-specific temperature annealing) and at 72 °C for 30 sec (elongation). Gene expression was normalized to the GAPDH housekeeping gene.

### miRNA expression analysis

Total RNA (10 ng) was reverse transcribed using miR-specific stem-loop RT primers (TaqMan MicroRNA Assays; Applied Biosystems, Thermo-Fisher) and components of the High Capacity cDNA Reverse Transcription kit (Life Technologies) according to the manufacturer’s protocols. Expression levels of individual miRNAs were detected by subsequent RQ-PCR using TaqMan MicroRNA assays (Life Technologies) and a Rotor Gene 3000 PCR System Corbett (Qiagen) using standard thermal cycling conditions in accordance with manufacturer recommendations. PCR reactions were performed in triplicate in final volumes of 30 µl, including inter-assay controls (IAC) to account for variations between runs. RT-PCR (TaqMan MicroRNA Assays; Applied Biosystems, Thermo-Fisher) was used to quantify the expression of has-miR-16-1, has-miR-15a and of has-miR-34a according to the manufacturer’s instructions (Table 2). To normalize the data for quantifying miRNAs, the universal small nuclear RNU38B (RNU38B Assay ID 001004; Applied Biosystems) as an endogenous control was used (Table 2)^59^.

The delta–delta Ct method was employed to calculate the fold change. In brief, each 15 μl of the reaction system contained 0.15 μl of 100 mM dNTPs with dTTP, 1 μl of MultiScribe Reverse Transcriptase (50 U/μl), 1.5 μL of RT buffer (×10), 0.1 μl of RNase inhibitor (20 U/μl), 6.25 μl of nuclease-free water, 5 μl of small RNA, and 3 μl of RT primer. Small RNAs are quantified by Qubit 3 fluorimeter (Life Technology). Thermal-cycling conditions were 30 min at 16 °C, 30 min at 42 °C, and 5 min at 85 °C. Each 20 μl of the reaction system for real-time quantitative PCR contained 1 μl of real-time primer, 1.33 μl of product from RT reaction, 10 μl of TaqMan Universal PCR Master Mix, and 7.67 μl of nuclease-free water. The reactions were performed in triplicate on a Rotor Gene 3000 PCR System Corbett for 10 min at 95 °C, followed by 40 cycles of 15 s at 95 °C and 1 min at 60 °C. Along with the Cq-values calculated automatically by the SDS software (threshold value = 0.2, baseline setting: cycles 3–15), raw fluorescence data (Rn-values) were exported for further analyses.

### Statistical analysis

Results were expressed as mean ± SEM from at least three independent experiments. The statistical significance of parametric differences among the sets of experimental data was evaluated by one-way ANOVA and Dunnett’s test for multiple comparisons. Statistical analysis of the mitotic index and reporter assays data was performed using the Fisher’s exact test.

## Electronic supplementary material

Supplementary figures
