# Supplementary material for: Etoposide-resistance in a neuroblastoma model cell line is associated with 13q14.3 mono-allelic deletion and miRNA-15a/16-1 down-regulation
Source: Sci Rep. 2018 Sep 13;8:13762. doi: 10.1038/s41598-018-32195-7 (PMC6137223; doi:10.1038/s41598-018-32195-7)
Supplement: Supplementary file 1 — Supplementary figures [file 41598_2018_32195_MOESM1_ESM.pdf]

Etoposide-resistance in a neuroblastoma model cell line is associated with 13q14.3 mono-allelic deletion and miRNA-15a/16-1 down-regulation

Barbara Marengo, Paola Monti, Mariangela Miele, Paola Menichini, Laura Ottaggio, Giorgia Foggetti, Alessandra Pulliero, Alberto Izzotti, Andrea Speciale, Ombretta Garbarino, Nicola Traverso, Gilberto Fronza and Cinzia Domenicotti.

Legends of Supplementary Figures

Figure 1 supplementary. Full-length blots/gels of proteins illustrated in Figure 1 (Bax, Bcl2 and β-actin), Figure 2 (p53 and β-actin), Figure 3 (p53-ser15 and β-actin) and Figure 6 (BMI-1, p16 and β-actin).

Immunoblots shown are representative of three independent experiments with essentially similar results. β-Actin is the internal loading control. The membranes were first hybridized with the anti-primary antibody and then with the anti β-actin antibody. After each hybridization, the chemiluminescence of both membranes was analyzed. Protein molecular weights are shown on the right of each blot.

Figure 2 supplementary. Acute etoposide exposure does not modify PUMA protein (α and β isoforms) expression levels in parental and HTLA-ER cells.

A) Protein levels of PUMA α and β in HTLA-230 and HTLA-ER cells untreated or treated for 24 hrs with 1.25 μM etoposide. Immunoblots are representative of three independent experiments with essentially similar results. β-Actin is the internal loading control. Histograms summarize quantitative data of protein level means, normalized to β-actin expression ± S.E.M of three independent experiments.

Fig 3 supplementary. Microarray scatter plot analysis of gene expression of HTLA-230 and HTLA-ER cells.

Each gene is represented by a dot differently colored according to the intensity of the gene expression. The genes whose expression is up-regulated in HTLA-ER in respect to HTLA-230 cells are colored in red; the genes with a similar expression are colored in yellow and the down-regulated genes in blue. The spots outside the green parallel lines represent the genes with a 2-fold variation

in the expression. Genes whose expression is similar are located inside this interval. The transcripts of the TP53 gene and of P53-related genes (ATM, CHK, ATK, BMI-1) are indicated as white circles.

Figure 4 supplementary. Parental and HTLA-ER cells have a similar amount of MDM2 protein and do not show MDM2 amplification.

A) Protein levels of MDM2 in HTLA-230 and HTLA-ER cells untreated or treated for 24 hrs with 1.25 μM etoposide. Immunoblots shown are representative of three independent experiments with essentially similar results. β-Actin is the internal loading control. Histograms summarize quantitative data of protein expression levels means, normalized to β-actin expression ± S.E.M of three independent experiments.

B) FISH analysis of HTLA-230 and HTLA-ER cells. Upper panels: nuclei of both HTLA-230 and HTLA-ER cells with two MDM2 and two CEP12 signals. Lower panels: metaphase of HTLA-230 with two chromosomes 12 displaying one MDM2 signal each close to cep 12 signal (purple arrow) and metaphase of HTLA-ER cells with one chromosome 12 displaying one MDM2 signal close to cep12 signal (purple arrow), one derivative chromosomes 12 displaying cep12 signal and one derivative chromosome 12 displaying MDM2 signal.

Figure 5 supplementary. The functional properties of mutant TP53 A161T protein are independent from the protein steady-state level in yeast and human cell reporter assays.

A) Representative western blot showing the level of wild-type and mutant (A161T) P53 proteins found in yeast cell lysates derived from functional assays performed at 30°C and 36°C in yLFM-P21-5'. PGK1 was used for normalization. Histograms summarize quantitative data of protein level

means, normalized to PGK1 expression of three independent experiments. *p<0.01 vs wild-type P53.

B) Representative western blot showing the level of wild-type and mutant (A161T) P53 proteins found in HCT116 TP53-/- cell lysates following transient co-transfection with the indicated P53 expression vectors and P21 promoter-derived reporter. β-actin was used for normalization.
Histograms summarize quantitative data of protein level means, normalized to β-actin expression of two independent experiments.**p<0.005 vs wild-type P53.

Figure 6 supplementary. Full-length blots/gels of proteins illustrated in Figure 3 supplementary

A) The membranes (M#1, M#2) were first hybridized with the anti-P53 Ab (DO1; left images) and then with the anti Pgk1Ab (right images). After each hybridization, the chemiluminescence of both membranes was analyzed in the same UVITEC acquisition; B) The membrane M#3 was first hybridized with the anti-P53Ab (DO1) and then with the anti β-act Ab; EV, empty vector. Protein molecular weights are shown on the right of each blot.

Since in the same WB other mutant P53 proteins, not relevant for this paper, were loaded, the images have been vertically cutted.

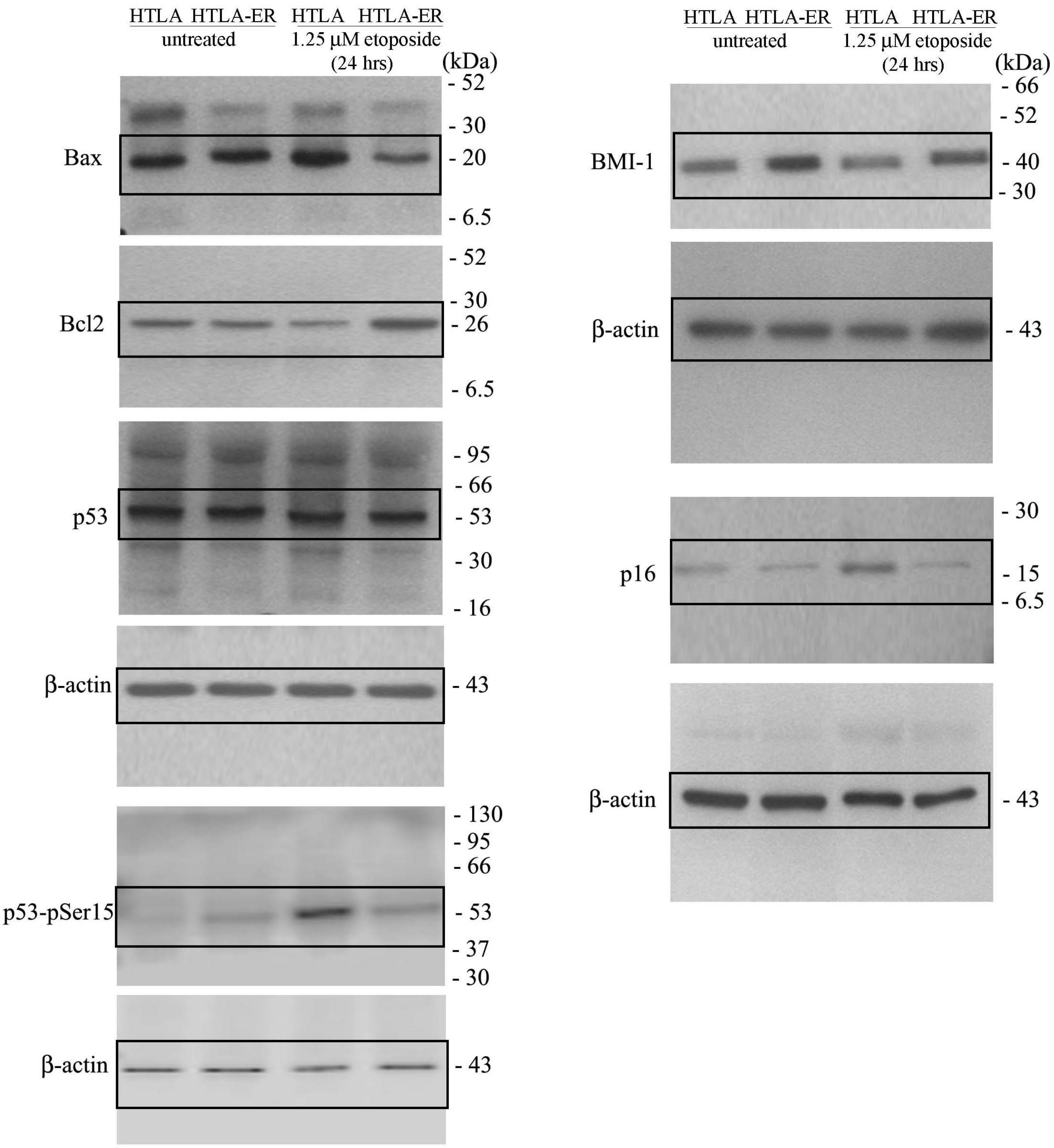
HTLA HTLA-ER
untreated
HTLA HTLA-ER
1.25 µM etoposide (24 hrs)
(kDa)
Bax
- 52
- 30
- 20
- 6.5
Bcl2
- 52
- 30
- 26
- 6.5
p53
- 95
- 66
- 53
- 30
- 16
β-actin
- 43
p53-pSer15
- 130
- 95
- 66
- 53
- 37
- 30
β-actin
- 43
HTLA HTLA-ER
untreated
HTLA HTLA-ER
1.25 µM etoposide (24 hrs)
(kDa)
BMI-1
- 66
- 52
- 40
- 30
β-actin
- 43
p16
- 30
- 15
- 6.5
β-actin
- 43

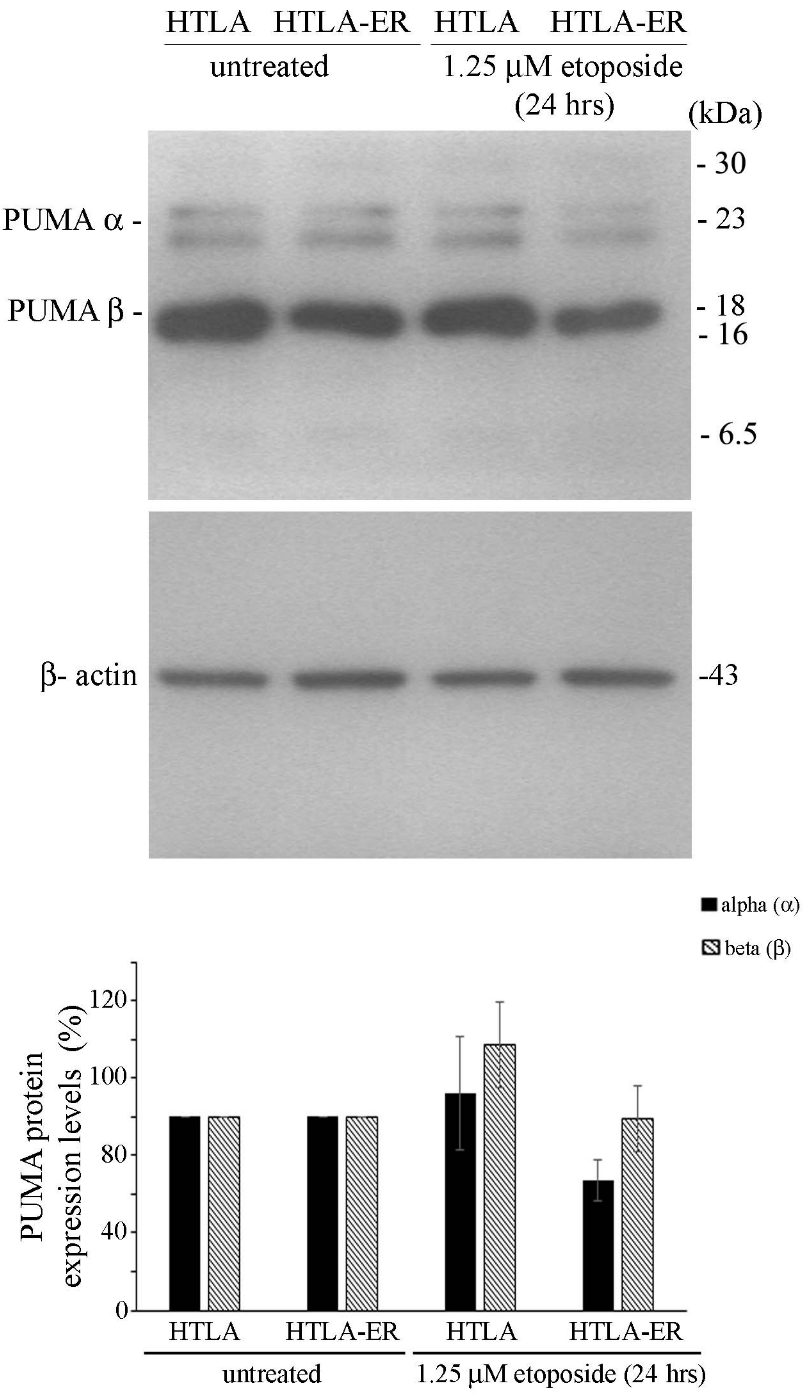
HTLA
HTLA-ER
HTLA
HTLA-ER
untreated
1.25 μM etoposide
(24 hrs)
(kDa)
- 30
PUMA α -
- 23
PUMA β -
- 18
- 16
- 6.5
β- actin
-43
alpha (α)
beta (β)
PUMA protein
expression levels (%)
120
100
80
40
0
HTLA
HTLA-ER
HTLA
HTLA-ER
untreated
1.25 μM etoposide (24 hrs)

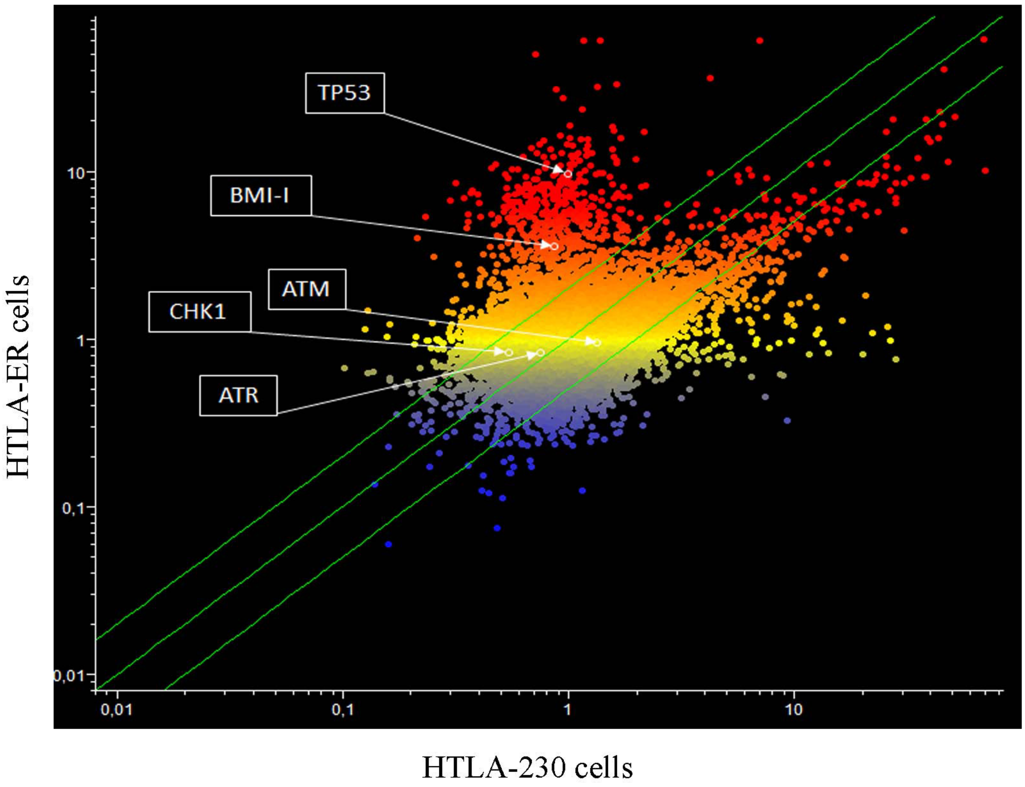
TP53
BMI-I
ATM
CHK1
ATR
10
1
0,1
0,01
0,01
0,1
1
10
HTLA-ER cells
HTLA-230 cells

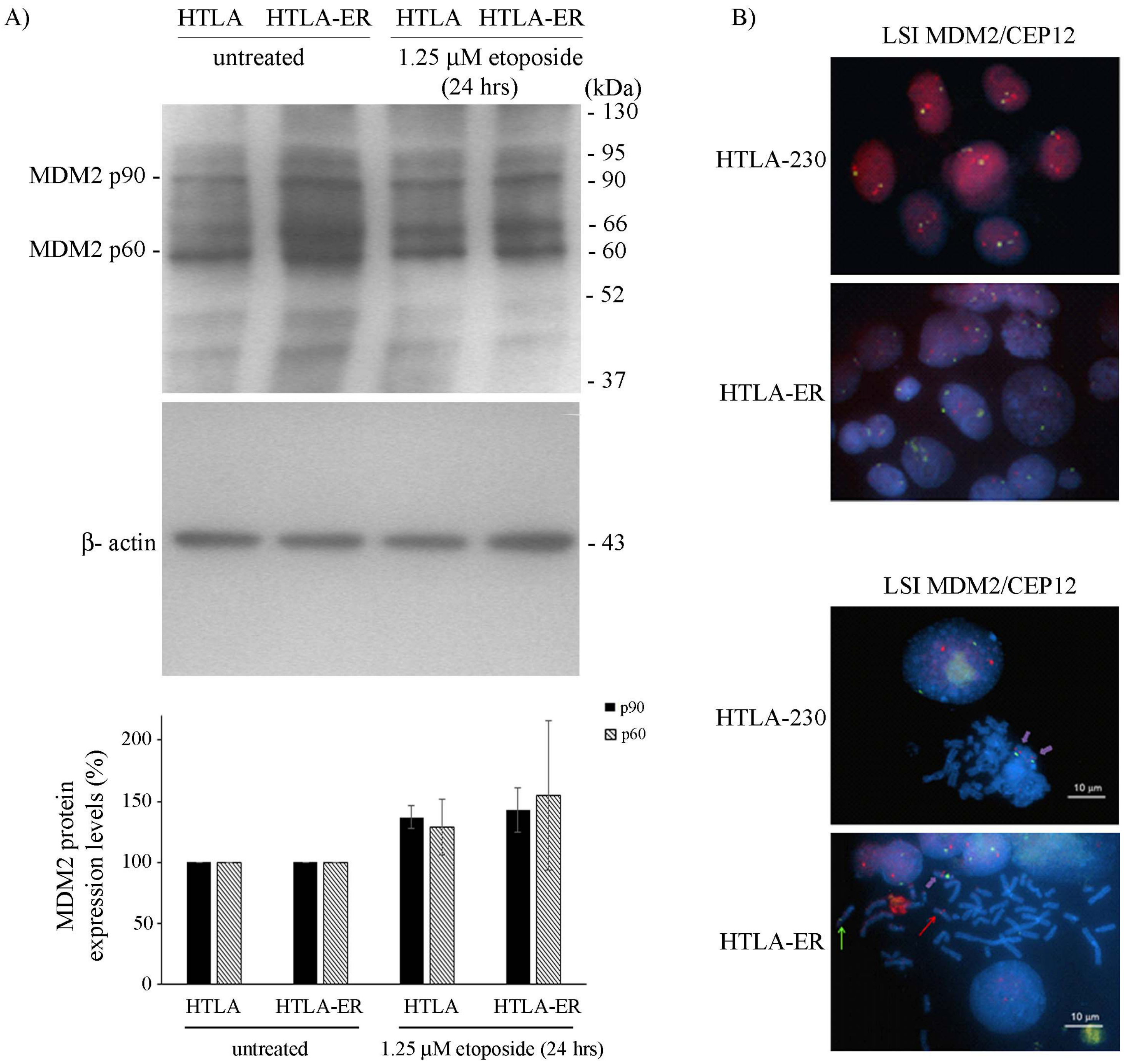
A)
HTLA
HTLA-ER
HTLA
HTLA-ER
untreated
1.25 μM etoposide (24 hrs)
(kDa)
- 130
- 95
- 90
- 66
- 60
- 52
- 37
MDM2 p90 -
MDM2 p60 -
β- actin
- 43
p90
p60
MDM2 protein expression levels (%)
200
150
100
50
0
HTLA
HTLA-ER
HTLA
HTLA-ER
untreated
1.25 μM etoposide (24 hrs)
B)
LSI MDM2/CEP12
HTLA-230
HTLA-ER
LSI MDM2/CEP12
HTLA-230
10 μm
HTLA-ER
10 μm

A)

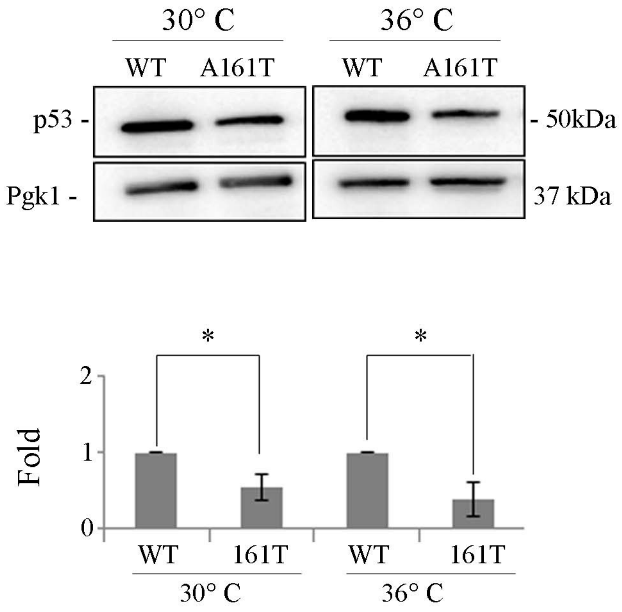

30° C
36° C
WT
A161T
WT
A161T
p53 -
Pgk1 -
- 50kDa
37 kDa
*
*
2
1
0
Fold
WT
161T
WT
161T
30° C
36° C

B)

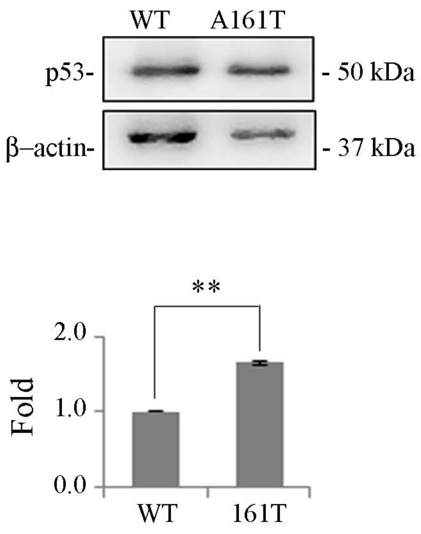

WT
A161T
p53-
β–actin-
- 50 kDa
- 37 kDa
**
2.0
1.0
0.0
Fold
WT
161T

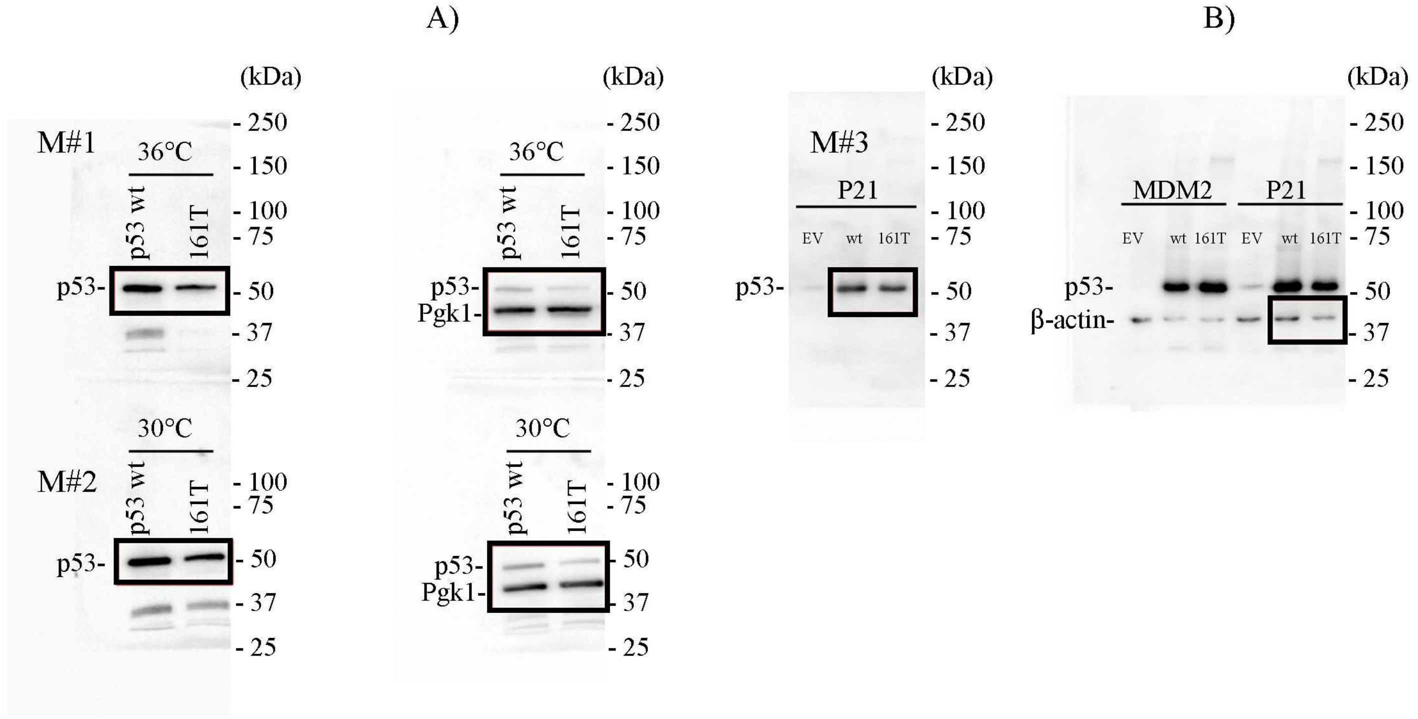

A)
B)
(kDa)
M#1
36°C
p53 wt
161T
p53-
250
150
100
75
50
37
25
M#2
30°C
p53-Pgk1-
M#3
P21
EV
wt
161T
MDM2
β-actin-
